# Unsupervised Text Segmentation Predicts Eye Fixations During Reading

**DOI:** 10.3389/frai.2022.731615

**Published:** 2022-02-23

**Authors:** Jinbiao Yang, Antal van den Bosch, Stefan L. Frank

**Affiliations:** ^1^Max Planck Institute for Psycholinguistics, Nijmegen, Netherlands; ^2^Centre for Language Studies, Radboud University, Nijmegen, Netherlands; ^3^KNAW Meertens Institute, Amsterdam, Netherlands

**Keywords:** text segmentation, eye movement, unsupervised learning, reading units, mental lexicon, computational cognition, cognitive unit

## Abstract

Words typically form the basis of psycholinguistic and computational linguistic studies about sentence processing. However, recent evidence shows the basic units during reading, i.e., the items in the mental lexicon, are not always words, but could also be sub-word and supra-word units. To recognize these units, human readers require a cognitive mechanism to learn and detect them. In this paper, we assume eye fixations during reading reveal the locations of the cognitive units, and that the cognitive units are analogous with the text units discovered by unsupervised segmentation models. We predict eye fixations by model-segmented units on both English and Dutch text. The results show the model-segmented units predict eye fixations better than word units. This finding suggests that the predictive performance of model-segmented units indicates their plausibility as cognitive units. The Less-is-Better (LiB) model, which finds the units that minimize both long-term and working memory load, offers advantages both in terms of prediction score and efficiency among alternative models. Our results also suggest that modeling the least-effort principle for the management of long-term and working memory can lead to inferring cognitive units. Overall, the study supports the theory that the mental lexicon stores not only words but also smaller and larger units, suggests that fixation locations during reading depend on these units, and shows that unsupervised segmentation models can discover these units.

## 1. Introduction

Language researchers may easily agree that an utterance comprises a sequence of “units,” but it is not easy to come to an agreement on what these units are. The units can be words, phonemes, morphemes, phrases, etc. from a linguistic perspective (Jackendoff, 2002); or unigrams, bigrams, trigrams, etc. from a statistical perspective (Manning and Schütze, 1999). In this paper, we take a *cognitive perspective* and aim to identify the cognitive units that play the role of building blocks in human language processing.

Words seem to be the most generally accepted units, perhaps because the spaces in written European languages steer us toward implicitly assuming that individual words are the most distinctive elements of sentences. Pollatsek and Rayner (1989) summarized ten key questions for the cognitive science of reading; nearly half of them are about words. Another case in point is that there are many models of visual word recognition, such as the Interactive Activation model (McClelland and Rumelhart, 1981), the Triangle model (Plaut et al., 1996), and the Dual Route Cascaded model (Coltheart et al., 2001). Even when considering sentence-level processing, researchers tend to take words as the basic units in their studies; this is the case for the classical studies relevant to the garden-path model which describes how the reader analyzes the grammatical structure of sentence from the serial input of words (Frazier and Rayner, 1982; Frazier, 1987), for the E-Z reader model which explains how the attributes of words guide eye movements during reading (Reichle et al., 1998), and for the discovery of the N400 component in brain activity which responds to semantically anomalous words (Kutas and Hillyard, 1980). Word units are also assumed for more recent studies such as those that map brain activity to processing of each word of a sequence (Brennan et al., 2012; Ding et al., 2016; Brennan and Hale, 2019) as well as studies that compare the statistical attributes of words in sentences with the cognitive and neural response to the words (Mahowald et al., 2013; Frank et al., 2015; Frank and Willems, 2017).

Though words are often used as psycholinguistic units, morphemes, which are defined as the smallest meaning-bearing units in a language (Chomsky, 1953), are usually the basic units in linguistic analysis. The central role of morphemes in linguistics also influenced some psycholinguists to consider the mental operations of morphologically complex words. In a recent review, Leminen et al. (2019) analyzed more than 100 neuroimaging studies of inflected words (e.g., walk-ed), derived words (e.g., dark-ness), and compounds (e.g., walk-man). As they summarized, most studies of the processing of derivational/inflectional morphology agree that such complex words are decomposed during processing; but studies of the processing of compound words show inconsistent results: some support the access of constituent morpheme units (Koester and Schiller, 2011; Fiorentino et al., 2014), some support the access of whole-word units (Stites et al., 2016), and some support mixed access of both (MacGregor and Shtyrov, 2013; Kaczer et al., 2015; Yang et al., 2020a).

In addition to subword units, the cognitive system can also make use of supra-word units. Some studies provide indications that supra-words such as frequent phrases and idioms (e.g., “I don't know") are stored in our long-term mental lexicon (Jackendoff, 2002; Bannard and Matthews, 2008; Arnon and Snider, 2010), implying that supra-words can be processed directly. Baayen (2007) has argued that the mental lexicon involves storage (of the wholes) and computation (of the combinatorial rules), and that they counterbalance each other. Yang et al. (2020a) also considered the counterbalancing, arguing that storing more supra-words in our mental lexicon could reduce the cognitive load of computation since larger units (e.g., “I am|going to" vs. “I|am|going|to") result in fewer processing steps (e.g., two retrievals + one combination vs. four retrievals + three combinations). Taken together, this diverse evidence shows that cognitive units exist at various linguistic levels, and that cognitive units have a wide range of possible lengths.

The flexibility of cognitive units implies that there is no clear or uniform perceptual salience of the units during reading, since a cognitive unit may be a sub-word or a supra-word that is not surrounded by two dividers (i.e., spaces), let alone the fact that in some writing systems (e.g., Chinese) there are no dividers for words. However, readers must be able to segment language input into cognitive units in order to access the meaning of the units and understand the input. Thus, our cognitive system must have a mechanism to quickly locate the cognitive units in language input for subsequent recognition. In fact, our eye movements in daily tasks may indicate the existence of this mechanism, since eye movements include many fixations which land neither randomly nor uniformly, but primarily on the targets of salience, information, or interest in the scene we see (Buswell, 1935; Henderson, 2011). So it is with reading: eye movements are controlled to skip some words, especially when the words are high-frequency function words (Rayner et al., 1982).

The flexibility of cognitive units also implies that it is hard for language learners to decide on the basis of perceptual cues whether or not a particular morpheme, word, or arbitrary string is a cognitive unit. Humans must have the ability to learn the cognitive units from their own experience, or in machine learning terms: unsupervised. To understand the human ability to learn and to identify the cognitive units, we need a model that is unsupervised and cognitively plausible. We here introduce the Less-is-Better (LiB) model (Yang et al., 2020b) as a candidate.

The LiB model is inspired by one intrinsic aspect of our nature: the principle of least effort. George Kingsley Zipf, who proposed the principle, explained it as “[the human agent] will strive to solve his problems in such a way as to minimize the total work that he must expend in solving both his immediate problems and his probable future problems.” (Zipf, 1949, p. 1). Limiting ourselves to purely cognitive tasks, we here interpret his words as:

*a. To reduce the number of* ***processing units*** *in* ***both current and prospective working memory.***

**Figure 1:**
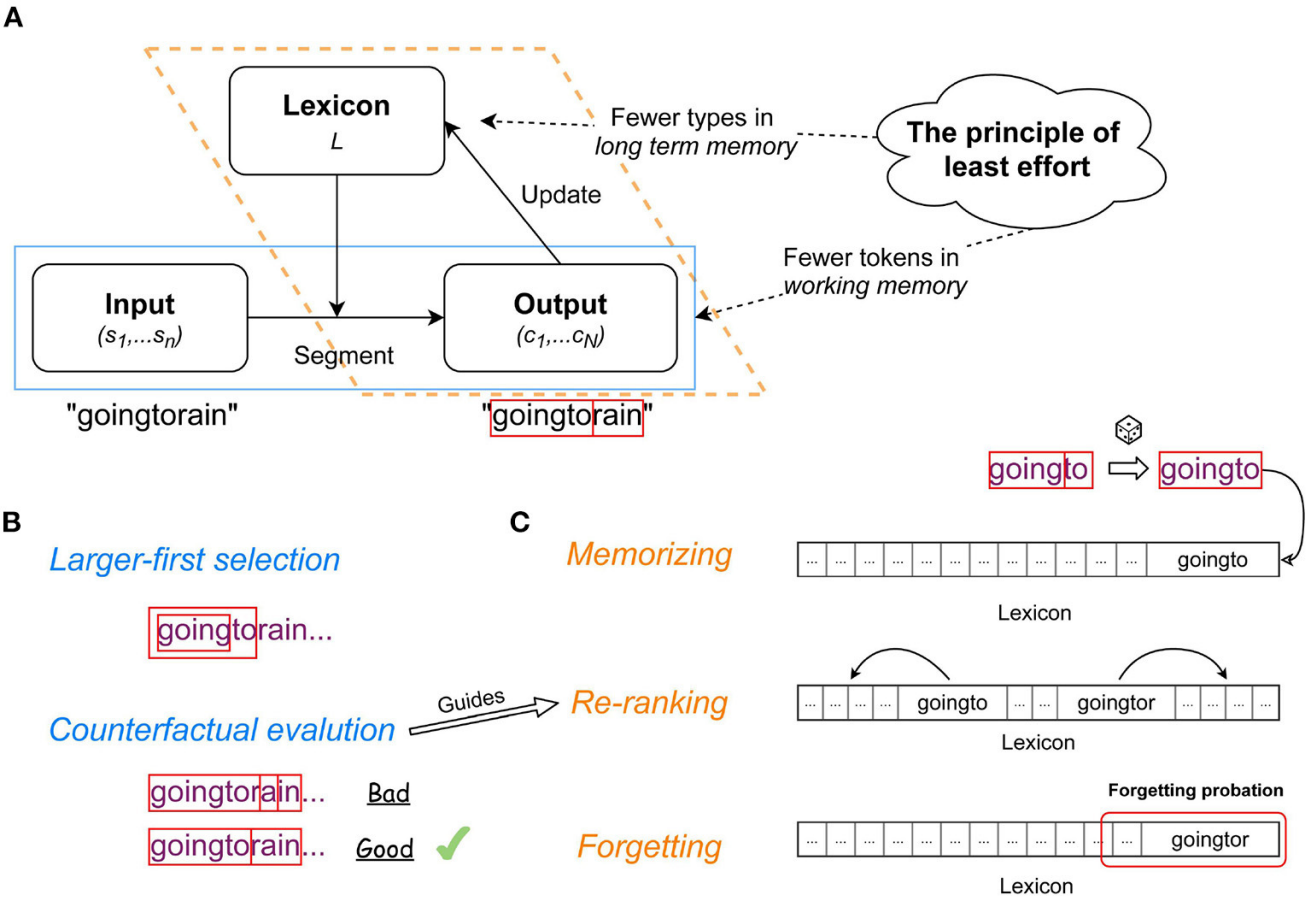
Illustration of the LiB model: **(A)** information flow in the LiB model; **(B)** the mechanisms in the text segmentation module; **(C)** the mechanisms in the lexicon update module.

The cognitive load refers to the demand not only on working memory, but also on long-term memory (see Figure 1A below), so we here extend the principle of least effort to:

*b. To reduce the number of* ***stored units*** *in* ***long-term memory.***

The LiB model regards the cognitive units as the language chunks that require the least effort during language processing, and the above two goals can be operationalized as: *a. to reduce the number of unit* ***tokens*** *in all potential texts*, and *b. to reduce the number of unit* ***types*** *in long-term memory (mental lexicon)*. There is a trade-off between the two goals. The former goal will prefer combining adjacent chunks into larger chunks, such as phrases, to reduce the number of tokens. If this process would be unrestricted, it would lead to units being so large as to represent the entire text with only one unit token. This would result in an extremely large lexicon memory that will not generalize to future use, as its units are unlikely to recur. To prevent this from happening, the latter goal will remove low-frequency chunk types from memory. The two goals counterbalance each other during learning and make the result in line with the least-effort requirement.

The current study aims to evaluate how similar the units segmented by unsupervised word segmentation models are to cognitive units. Although we lack a gold standard for cognitive units, eye movements during reading, specifically the eye fixations, may provide information about them. Taking words as units of analysis of eye-tracking data, studies have reported that fixation positions frequently fall at (or close to) the center of a word when the word is fixated only once (Rayner et al., 1996; Li et al., 2011; Paterson et al., 2015), but some words are fixated more than once (Rayner and McConkie, 1976; Hyönä and Olson, 1995; Kliegl et al., 2004; Cop et al., 2017), while some short words are not fixated upon (Kerr, 1992; Brysbaert and Vitu, 1998). Taking multiword sequences as units of analysis of eye-tracking data, formulaic sequences (e.g., “as a matter of fact") get fewer fixations than non-formulaic sequences (e.g., “it's a well-known fact”) (Underwood et al., 2004). In light of these empirical findings, we hypothesize that eye fixations are a proxy to the location of the cognitive building blocks of the text, that is, the cognitive units.

Our main goal in the current study is not to predict or explain eye fixations, but to validate the model as a cognitive model by quantifying its ability to predict eye fixations. The current model aims to be as simple as possible by using only unannotated text. Therefore, in this study, we will not include properties that can improve fixation prediction but are outside the scope of the model (e.g., semantics).

We use the units segmented by LiB in a corpus to predict the locations of the eye fixations in the same or a different corpus. If the LiB units indeed predict eye fixations, this suggests both that the LiB units are similar to the cognitive units and that the cognitive units are located by the eye fixations. In other words, cognitive units may be considered a latent factor driving both eye fixations and the discovery of units by LiB, and the extent to which the LiB units predict eye fixations reflects their plausibility as cognitive units. Then we evaluate the similarity between the LiB units and the hypothesized cognitive units during human reading by comparing the eye fixations predicted by LiB with the observed eye fixations extracted from an eye-movement corpus. As the design and the training of the model are independent of the eye movements (i.e., the model is not fitted on the eye-tracking data), any overlap found between model predictions and eye movements is caused by properties of the model itself and not by spurious patterns discovered in the eye-movement data.

Two other segmentation models are also evaluated for comparison: Chunk-Based Learner (McCauley and Christiansen, 2017), and Adaptor Grammar (Goldwater et al., 2009). We also compare to two word-based baselines: one that assumes the cognitive units are equal to words, and one that assumes the cognitive units are determined by the word length. The models are introduced in more detail below. In the comparisons, we will demonstrate that the segmentation models outperform the baselines, and show the advantages of LiB in various aspects.

## 2. Methods

### 2.1. The Less-is-Better Model

The information workflow of the LiB model consists of an interaction loop between the text segmentation module (blue box with solid line; Figure 1A) and the lexicon update module (orange box with dotted line; Figure 1A). We briefly characterize the model; more detail is given in Yang et al. (2020b). The model has a lexicon *L* which is an ordered set of unit types *u*. In each epoch, the segmentation module (Figure 1B) segments the input, which is a sequence of symbols (*s*_1_, *s*_2_, …, *s*_*n*_) (in the current simulations, symbols are characters, excluding spaces) into a sequence of a minimal number of unit tokens (*u*_1_, …, *u*_*N*_), where each *u* token is a subsequence of the input; *u* = (*s*_*i*_, …, *s*_*j*_), and each *u* is in the current *L*. The update module (Figure 1C) then updates *L* according to the output (*u*_1_, …, *u*_*N*_), meaning that some new unit types are created and added to *L* to decrease the number of tokens in future inputs, and some current unit types are removed to decrease the number of types in *L*.

To reduce the number of *u* tokens during the segmentation, if *u* types of different sizes in *L* match the current input, the largest *u* type has the priority to be selected as the *u* token (*Larger-first selection*; Figure 1B). Then LiB evaluates the *u* token by segmenting the following input and counting the segmented tokens. LiB segments the current input as if the largest *u* type does not exist (*counterfact*) so the second-largest u type will also be evaluated. In case the largest *u* type causes more *u* tokens in the input than the second-largest *u* type, the largest *u* type is evaluated as *Bad* (otherwise as *Good*) and the second-largest *u* type is selected instead (*Counterfactual evaluation*; Figure 1B).

*L* is empty at the beginning, and all the symbols *s*_1_, …, *s*_*n*_ in the input are unknown to the model. Those symbols will be memorized as the first batch of new *u* types in *L*. Adjacent *u* tokens in the input can become a larger *u* token by concatenation of the two original tokens, and the adjacent larger *u* tokens can become an even larger *u* token. LiB randomly samples the combinations of segmented *u* tokens and memorizes the sampled combinations as new *u* types (*Memorizing*; Figure 1C). These new *u* types go to *L* immediately and can be used for further segmentations and combinations, so LiB learns online. The sampling strategy achieves similar results as tracking the frequencies of each *u* type and dropping the low-frequency ones, since the *u* types with higher frequencies are more likely to be sampled. However, compared to frequency tracking, LiB's sampling strategy consumes markedly less resources of memory and operation.

Although no statistical information of the *u* types is recorded, LiB indicates a *u* type's likelihood of being a cognitive unit by the type's rank in the Lexicon. A newly memorized *u* type is appended to the end of *L*, which means it has the lowest likelihood of being a cognitive unit, because the new *u* type might merely be an accidental concatenation of two *u* tokens. Besides the memorizing order, the order of *L* also depends on *Chunk evaluation*: after the evaluation, a *Good* *u* is moved forward and a *Bad* *u* is moved backward in *L* (*Re-ranking*; Figure 1C).

The *Re-ranking* pushes the chunks *u* that were evaluated as *Bad* as well as very infrequent chunks (that never had the opportunity to be evaluated) backward in *L*. This means the end of *L* contains not just newly memorized *u* but also junk *u* (infrequent *u* and *Bad* *u*). To clean up only the junk *u*, all *u* at the end of *L* enter a probationary period. In case a *u* was evaluated as *Good* during the probation, its probation is canceled; otherwise, the chunk is removed from *L*. By such a mechanism (*Forgetting*; Figure 1C) LiB can reduce the number of *u* types and keep a small size *L*.

### 2.2. Other Models for Evaluation

Firstly we introduce a frequentist computational model named Chunk-Based Learner (CBL; McCauley and Christiansen, 2019), which aims to simulate human incremental language acquisition. CBL also has its cognitive basis: frequency-based learning. In detail, CBL processes naturalistic linguistic input as sequences of chunks. Initially, each word is a chunk. Then CBL calculates the backward transitional probabilities (BTPs) between the chunks. If the BTP of a chunk-pair rises above the average of all tracked BTPs, the chunk-pair will be grouped as a new chunk and be replaced by the new chunk in further processes. CBL in this way implements the incremental learning of multi-word units. Some words will not be combined into larger chunks, and thus the lexicon of CBL will contain both word units and multi-word units.

Bayesian models can be seen as an alternative to frequentist models, and the “Bayesian coding hypothesis" also argues that humans behave Bayesian (Knill and Pouget, 2004). Adaptor Grammar (AG; Johnson et al., 2007) is a word segmentation model based on a Bayesian framework. Like the other models we compare, it aims to segment the tokens from the input in an unsupervised way. The AG model represents each input sequence from the corpus as a multi-level tree structure with a predefined number of levels. Although different trees can represent the same sequence, AG assumes there is an optimal tree. The Hierarchical Dirichlet Process, which is a nonparametric Bayesian approach to group the observed data hierarchically (Teh et al., 2006), is used to find the *optimal* trees that fit the input sequences. Not with standing AG is usually used for word segmentation, syllabification, and other linguistic applications (Johnson et al., 2007; Johnson, 2008; Johnson and Goldwater, 2009; Zhai et al., 2014), in the current study we investigate whether the unsupervised nature of the model can help to discover the cognitive units.

Besides the segmentation models that can generate non-word units, we set two baselines that are completely word-based. The first baseline (*Word-by-Word*) simply assumes that the cognitive units are equal to words. As we mentioned above, words are the commonly accepted units in many studies so it is worth investigating whether words or the model-produced cognitive units can better predict eye fixations.

Another baseline (*Only-Length*) implements the assumption that the number of fixations on a word is determined by the length of the word. Different from the *Word-by-Word* baseline, the *Only-Length* baseline uses the knowledge of observed eye fixations. *Only-Length* groups the words with the same number of letters together and shuffles the numbers of fixations within each group. Only the distributions related to the word lengths persist in this baseline so the prediction will not be influenced by frequency, morphology, position, or other non-length information.

### 2.3. Eye Fixation Data

The eye fixation data is extracted from the Ghent Eye-Tracking Corpus (GECO) corpus^1^ (Cop et al., 2017). GECO contains three sets of eye-tracking data: 14 English monolinguals reading the English novel *The Mysterious Affair at Styles* by Christie (2008) (monolingual set); nineteen Dutch (L1)–English (L2) bilinguals reading the same novel (L2 set); and the same bilinguals reading the Dutch translation of the novel (title in Dutch: *De zaak Styles*) (L1 set). The English monolingual group read the full English novel and the bilingual group read either the first half of the novels in English and the second half in Dutch, or vice versa. For the evaluation in the current study, we discard the L2 set since it is not native-language reading.

The GECO datasets provide two types of eye fixation data: *first-pass fixation count* and *total fixation count*. The first-pass fixation includes only the initial reading (until any fixation on another word) within each word and the total fixation includes also the re-reading (after regression) within each word. Most of the regressions reflect post-lexical language processing (Reichle et al., 2009), and others may reflect oculomotor error or difficulty associated with the identification of words (Vitu and McConkie, 2000). These processes are beyond the scope of the segmentation models we evaluated, since the segmented cognitive units are for planning what to process rather than *post-hoc* adaptation. That being so, we evaluate only the first-pass fixation count.

### 2.4. Corpora

Both the English and the Dutch GECO corpora are used for model training in the current study. Since the material presented to the participants are in multiple lines, and the last word in a line and the first word in the next line are too far apart to be perceived as a cognitive unit, we break any sentence that appears across different lines into separate sequences. Two other corpora also serve as training material but only in the generalizability test of the models. One of them is Corpus of Contemporary American English (COCA; Davies, 2008). We used a sample dataset of COCA which is free for the public^2^. Although the sample dataset is only a small part of the complete COCA corpus, it is more than 100 times larger than the English material in GECO. The other additional training corpus is SoNaR (Oostdijk et al., 2013), a 500-million-word reference corpus of contemporary written Dutch from a wide variety of text types. The complete corpus is very large so we selected the *book* subset of SoNaR. The corpus sizes are shown in Table 1.

**Table 1:** The corpora statistics after preprocessing.

**Language**	**Corpus**	**Sentences**	**Word tokens**	**Word types**
English	GECO	13,491	57,170	5,316
	COCA (sample)	1,745,060	9,451,421	140,553
Dutch	GECO	13,407	60,836	5,859
	SoNaR (books)	3,308,337	22,802,170	272,865

The text from all corpora was converted to lowercase. Dutch characters with accents (*diacritical characters*) were replaced by their unaccented counterparts (e.g., ë → e). All punctuation (except the apostrophe as a part of possessive) was used as a divider between the input sequences and then were replaced by a space. Finally, all sentences have a space added at the end to make sure that all word tokens end with a space.

### 2.5. Evaluation

To evaluate the units segmented by the different models against the eye fixation counts on each word from GECO, we predict the eye fixation count from the segmentation models and from the word-based baselines, and then compare the predicted eye fixation counts per word with the observed eye fixation counts.

For the segmentation models, the eye fixation counts are predicted in the following procedure:

1. **Training the models:**
  - **The LiB model**: In each training epoch, a 200-sentence batch is randomly extracted from the corpus text (batch-based update in LiB reduces the computing cost) and then fed into the model. When training on GECO, which is rather small, the batch extraction is with replacement and the training stops when the number of input encoding bits^3^ no longer decreases. When training on the large-scale corpus, the batch extraction is without replacement, and the training will stop when there is no training material left. The hyperparameter settings in the current study follow the previous LiB study (Yang et al., 2020b) except the setting of *probation period*^4^.
  - **The CBL model**: Different from LiB and AG which regard the input as a sequence of characters, CBL regards the input as a sequence of words, so it preprocesses the input into words based on the spaces. There is no change in the training stage of the original code of McCauley and Christiansen's (2019) implementation^5^.
  - **The AG model**: The simplest grammar tree in AG starts with characters, then processes words, and then sentences. The model tends to under-segment without an intermediate level of collocations (Johnson and Goldwater, 2009), so the AG grammar tree used in the current study is: character(s) → word, word(s) → collocation, collocation(s) → sentence. Besides the design of the grammar tree, we also follow the hyperparameter settings of Johnson and Goldwater's (2009) experiment^6^.
2. **Segmenting the text into units:**
  - **The LiB model**: Each sequence of the GECO corpus is fed to the trained model to be segmented into the units existing in the trained lexicon. The segmentation is guided by *Larger-first selection* and *Counterfactual evaluation*. No new cognitive units will be memorized in this stage.
  - **The CBL model**: The original implementation simulates children's incremental learning so the lexicon is empty at the start of training. This means a group of words may be a unit at the end of segmenting the GECO corpus but not at the beginning. To keep the segmentation consistent in the test material, as in the other models, our implementation of CBL learns the training corpus thoroughly and then segments the test corpus again with a fixed lexicon.
  - **The AG model**: AG learns the parsing rules during training. When the model applies the rules to the test corpus, each sequence will be processed into a hierarchical structure which contains the *character* level, the *word* level, the *collocation* level, and the *sentence* level. We extracted the units at the *word* level (the *AG-word* units) and at the *collocation* level (the *AG-collocation* units).
3. **Predicting the number of fixations on each word**:We assume that reading is based on the cognitive units and the fixation positions are the centers of the cognitive units, at least if the entire unit is within the perceptual span. We ignore the perceptual span for now since we want to evaluate the models totally free of prior limitations. We calculate the predicted number of fixations on a word as the number of cognitive units centered on the word. For example, the predicted fixation position of the unit *I have* is between *h* and *a*, so the predicted fixation number is zero on the word *I* (we can also say *I* is skipped in this case) and one on the word *have*. The predicted fixation number of the word *neuroscience* is two if it is segmented into *neuro* and *science*.
  To investigate the possible effect of perceptual span limitations, we also evaluate LiB while considering the perceptual span. We set different upper limits on the unit length in the model and expect there to be a maximum length that is optimal for the prediction of fixation counts and that equals the perceptual span.

For the word-based baselines, the eye-fixation numbers are predicted differently:

- The *Word-by-Word* baseline predicts exactly one fixation on each word;
- The *Only-Length* baseline groups the words with the same length together and randomly shuffles the observed number of fixations on the words within each group. Hence, the predicted number of fixations of a word is actually the observed number of fixations of another word with the same length.

The last step in the evaluation is comparing the predicted number of eye fixations with the observed number of eye fixations on each word. The F1 metric (Equation 1) is commonly used for evaluating a binary classification model based on the predictions made for the positive class (Van Rijsbergen, 1979).


(1) $$\begin{array}{l} \text{F1}=\frac{\text{True positive}}{\text{True positive}+0.5\times \left(\text{False positive}+\text{False negative}\right)} \end{array}$$

However, both the observed number and our predicted number of eye fixations are not binary, in other words, the metric must work for multi-label data. Because of the very imbalanced distribution of the fixation counts, we choose weighted F1 as the measure of prediction accuracy^7^. The weighted F1 calculates the binary F1 metric, which is shown by Equation 1, for each label (fixation count), and finds their average weighted by the number of true instances for each label.

## 3. Results

### 3.1. Qualitative Comparison

Firstly we provide some segmentation examples generated by the different models (Table 2). In general, short and frequent collocations tend to form individual units (e.g., *to do*), and some of those collocations are not in a syntactic phrase (e.g., *i was*); long words tend to be divided into subword units (e.g., *uitnodigde* segmented as *uit|nodig|de*). Each model has its own characteristics: the CBL model learns no subword units; the AG-word model always over-segments the text; the LiB units and the AG-collocation units are generally similar.

**Table 2:** Segmentation examples from different models in English and Dutch.

		**Language**	
**Sample**	**Model**	**English**	**Dutch**
1	Input	i was trying to make up my mind what to do	was ik nog aan het overleggen wat ik zou gaan doen
	LiB	i was |trying to |make |up |my mind |what |to do	was ik |nog |aan het |over|leggen |wat ik |zou gaan |doen
	CBL	i |was trying |to make |up |my mind |what |to do	was |ik nog |aan |het overleggen |wat |ik zou gaan |doen
	AG-word	i |was |try|ing |to |make |up |my |mind |what |to |do	was |ik |nog |aan |het |over|leg|gen |wat |ik |zou |gaan |doen
	AG-collocation	i was |trying to |make |up |my mind |what |to do	was ik |nog |aan het |over|leggen |wat ik |zou gaan |doen
2	Input	and it ended in his inviting me down to styles to spend my leave there	en het eind van 't liedje was dat hij mij uitnodigde mijn verlof door te brengen op styles
	LiB	and it |ended |in his |invi|ting |me |down to |styles to |sp|end |my |leave |there	en |het eind |van 't |li|e|d|je was |dat hij mij |uitnodig|de |mijn |verlof |door te brengen |op styles
	CBL	and |it ended |in |his inviting |me |down |to |styles to spend |my |leave |there	en |het eind |van |'t liedje |was |dat hij |mij uitnodigde |mijn verlof |door |te brengen |op styles
	AG-word	and |it |end|ed |in |his |invit|ing |me |down |to |styl|es to |spend||my |leav|e |there	en |het |eind |van |'t |lie|d|je |was |dat |hij |mij |uit|nodig|de |mijn |ver|lo|f |door |te breng|en |op |styles
	AG-collocation	and |it |ended |in his |inviting |me |down to |styles to |spend my |leave |there	en |het eind van |'t |lied|je |was |dat |hij mij |uitnodig|de |mijn |verlof |door |te brengen |op styles

### 3.2. Unit-Length Comparison

**Figure 2:**
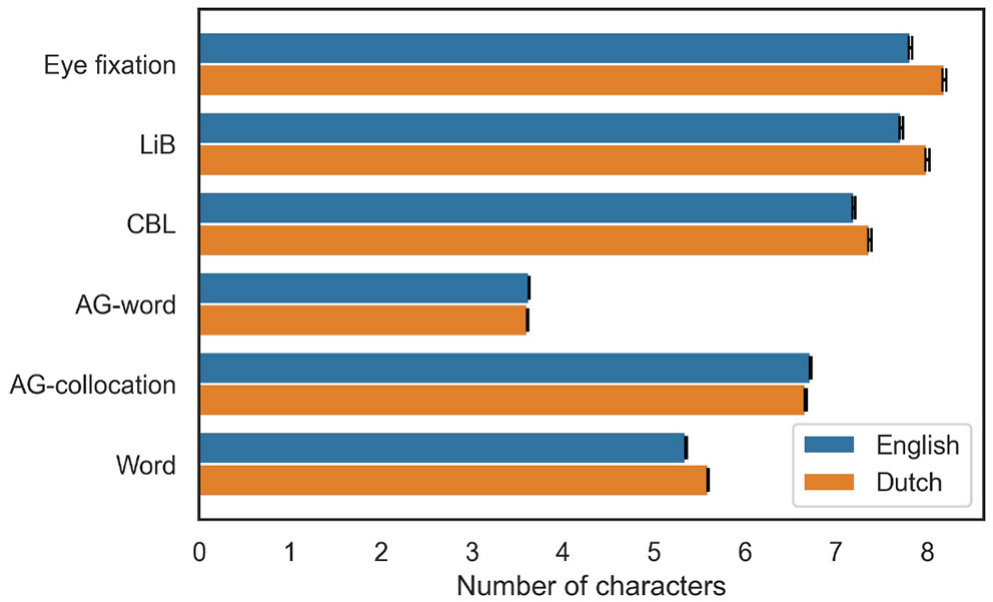
The average token lengths of the observed eye fixation units, the model-segmented units, and linguistic words in English and Dutch texts. The error bars represent 99% confidence intervals.

The segmentation examples show the models' output are markedly different from each other even though they are all unsupervised models. To investigate in more detail how the models' outputs differ and relate to eye fixations, we first look at the average of the unit token lengths of the models and the observed eye fixations. The GECO dataset does not provide the locations but only the number of eye fixations in any word, so we do not know every interval between each two eye fixations. Instead, we infer the locations of eye fixations from their counts in each word and then calculate the lengths of the eye fixation units by assuming eye fixations are located in the middle of the units. Figure 2 shows that the average unit length of the observed eye fixations is clearly longer than the average length of space-delimited words, and is close to the average unit length of LiB. Among the unsupervised models, only AG-word shows even shorter unit length than the linguistic words. Moreover, Dutch units are in general slightly longer than English units, except in the AG models.

### 3.3. The Distributions of Predicted and Actual Fixation Counts

**Figure 3:**
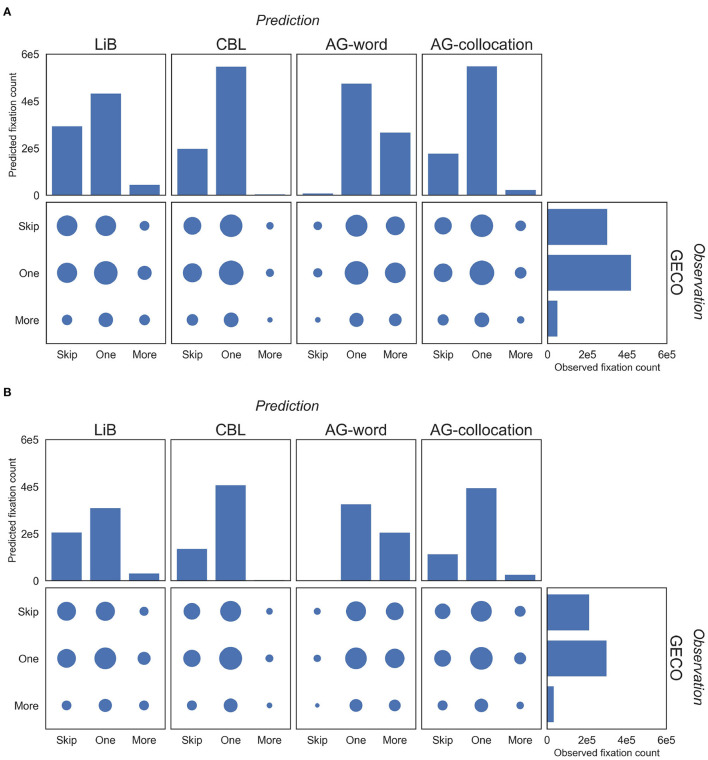
Distributions of the counts of the (predicted) eye fixations on the English **(A)** and Dutch **(B)** corpora. Firstly we define three labels of the fixation counts (“*Skip”*: 0, “*One”*: 1, and “*More”*: >1). The histograms present the distribution of three labels; specifically, the vertical histograms present the predictions of the models and the horizontal histogram presents the observations in GECO. The scatter plots present the confusion matrix between the model predictions and the GECO observations; the surface area of each circle indicates the item count of the matching instance.

Next, we predicted the number of eye fixations on each word token from the segmentation of the models. We display the joint distribution of the predictions and observed eye fixations (Figure 3). The CBL model's output has only very few subword units (indicated by *More*, meaning more than one predicted fixation on the word). In fact, CBL itself does not output any subword units, but there are some hyphenated tokens in GECO (e.g., *forty-five*), which are processed as multiple words by the models while GECO (and so the evaluation) regards them as single words. AG-word has only very few supraword units (indicated by *Skip*, meaning no fixation at the word). Compared to the distributions of other models' predictions, the distribution of LiB's prediction is most similar to the distribution of observed fixations on both the English and the Dutch dataset. Furthermore, the surface area of the circle in the confusion matrix (Figure 3) shows that *One* (exactly one fixation at the word) predictions match the observed data most often for all models, and that *More* predictions match the observed data the least.

### 3.4. The F-Scores of Model Predictions

The unit lengths and fixation distributions displayed above (Figures 2, 3) provide an overview of the differences between the predicted eye fixations by the different models and the observed eye fixations. Next, we quantitatively evaluate the similarity between the predicted and observed eye fixations by their weighted F1 scores.

Table 3 shows that three of the four segmentation models outperform the word-based baselines in the eye-fixation prediction tasks. The *Only-Length* baseline, which predicts by only the word length, is better than the *Word-by-Word* baseline, and close to the segmentation models. Out of the four models, LiB and AG-collocation produce the best predictions and AG-word produces the worst predictions, worse than the word-based baselines.

**Table 3:** Evaluations of models/baselines in different languages.

**Model**	**English**	**Dutch**
LiB	53.06	**51.87**
CBL	52.20	50.04
AG-word	30.10	28.95
AG-collocation	**53.35**	51.45
Word-by-Word	38.32	38.68
Only-Length	50.82	50.57

*All the scores are the weighted F1 metric between the predicted eye fixations and the observed eye fixations.*

*Bold values indicate the highest score across models*.

### 3.5. The Effect of Unit-Length Limitation

**Figure 4:**
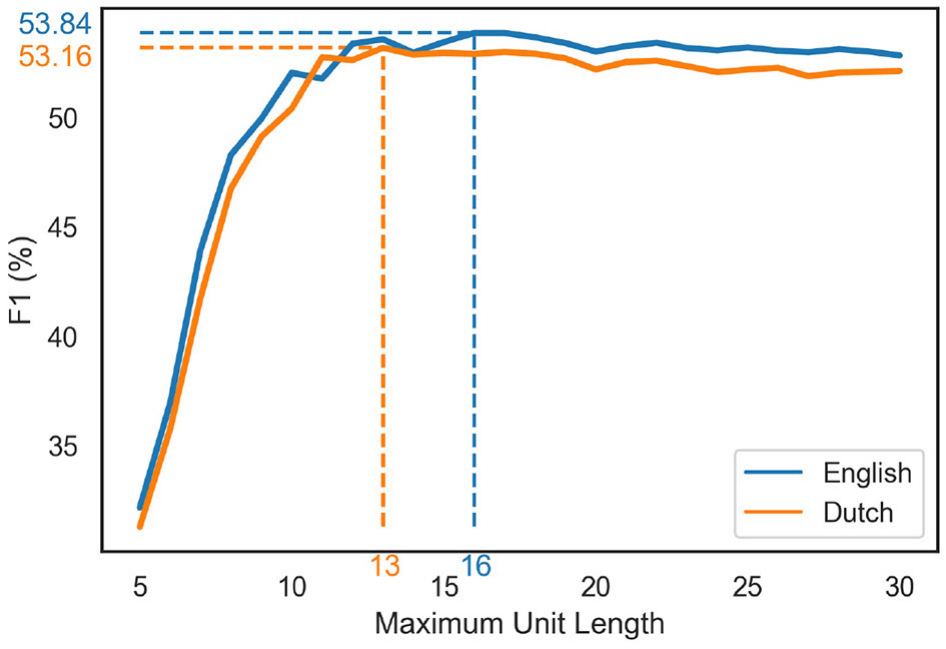
The prediction scores with different LiB unit length limitations. The blue/orange dotted lines indicate the peak scores and the corresponding maximum unit length in the English/Dutch simulations, respectively.

Next, we evaluate LiB under different limitations of unit length, which captures possible perceptual limitations. The sharp rise of the prediction scores with increasing maximum length quickly levels off (Figure 4). The prediction scores even slightly decrease after the peaks: the optimal maximum unit lengths (indicated by the arrows in Figure 4) are 16 for English (F1 = 53.84) and 13 for Dutch (F1 = 53.16).

### 3.6. Training on Non-GECO Corpus

The eye fixation data are from the GECO corpora, which are also the model training corpora for the results above. To test the generalizability of the models, we evaluate the models trained on the non-GECO large scale corpora and compare the results with the models trained on the GECO corpora^8^. Table 4 shows that training on the non-GECO corpora improves the prediction of eye fixations compared to training on the GECO corpora themselves. This is the case for both LiB and CBL, although the predictions by LiB remain the most accurate. CBL shows high time efficiency since its training is based on words rather than characters (as in LiB and AG). However, CBL, compared with LiB, shows a higher *relative* increase in training time with the same increase of the training materials. Moreover, CBL tracks the frequencies of all words and the backward transitional probabilities of all word pairs, which causes the sharp growth of the lexicon on the large corpora.

**Table 4:** Comparison of training times and F1 scores between different models and different training corpora.

**Model**	**Training corpus**	**English**			**Dutch**		
		**Training time**	**Lexicon size**	**F1 score (%)**	**Training time**	**Lexicon size**	**F1 score (%)**
LiB	GECO	2 min 31 s	15,867	53.06	2 min 38 s	17,525	51.87
	COCA/SoNaR	24 min 51 s	97,872	**53.46**	72 min 5 s	143,665	**53.72**
CBL	GECO	1 s	29,268	52.28	1 s	33,248	50.04
	COCA/SoNaR	1 min 24 s	2,051,239	53.30	3 min 23 s	3,782,605	51.71

*COCA/SoNaR means the training corpus is COCA for the English task and SoNaR for the Dutch task. The Lexicon size of CBL is the sum count of its stored unigrams and backward transitional probabilities between the unigrams.*

*Bold values indicate the highest score across models*.

## 4. Discussion

In this study we have shown how to predict eye fixations on text by unsupervised segmented cognitive units. Conversely, we evaluate these units by their predictions of eye fixations. In particular, we tested three segmentation models: the LiB model, the CBL model, and the AG model. We also compared them with two word-based baselines: assuming that reading is word by word; and assuming that we can predict the number of fixations on each word by the length of the word. Firstly, we found eye fixations can be better explained by the cognitive-unit-based models than by the word-based models, and both the LiB and AG models predicted the fixations best among the cognitive-unit-based models. Secondly, the predictions are robust between two languages (English and Dutch). Lastly, we found the LiB and CBL models can predict eye fixations on a different corpus, and large-scale training material improves the prediction.

### 4.1. From Word-Based to Cognitive-Unit-Based Reading Theories

The evaluations in the current paper show that eye fixations during reading can be predicted by unsupervised text segmentation models (Figure 4; Tables 3, 4). These results suggest a cognitive-unit-based eye-movement planning in the oculomotor system. Eye fixation during reading is not arbitrary nor guided by purely orthographic cues such as spaces and punctuations, so the reader's oculomotor system must plan the fixations by using both orthographic cues in the text and top-down knowledge.

Traditional theories of fixation-planning regard words as reading units. To explain the fixations which are not word by word (*fixating words more than once* or *skipping words*), it is usually assumed that a word's lexical attributes (e.g., frequency, predictability, and length) can help to decide whether to refixate or skip the word. An example of such a theory is the E-Z reader model (Reichle et al., 1998, 2009; Reichle and Sheridan, 2015), which is one of the most popular eye movement models. It assumes that our visual system can preview the text to the right of the current fixation and make use of the lexical attributes of the next word to plan the next fixation position.

Different from the traditional word-based theories, we regard *cognitive* units as reading units and assume that most of the first-pass fixations are at the center of each reading unit (we here ignore the limitation of perceptual span for simplicity and will get back to it later). Based on this assumption, the fixation-planning task may be approximated as a cognitive-unit segmentation task, just as we did in this study. The cognitive-unit-based predictions (from the *LiB, CBL*, and *AG* models) are generally better than the word-based predictions (*Word-by-Word* and *Only-Length*) (Table 3). *Word-by-Word* assumes reading proceeds with one fixation per word, but the baseline's poor performance undermines this assumption. *Only-Length* assumes that the number of fixations on a word is determined solely by the word's length. It scores higher than *Word-by-Word* showing that longer words tend to be fixated more often (which is already well known). Importantly, *Only-Length* predictions are still worse than the cognitive-unit-based predictions, even though its predictions use the distributions of observed eye fixation, which the segmentation models are ignorant to. To sum up, the cognitive-unit-based approaches can outperform the word-based approaches with less information and even when allowing for unrealistically long units (i.e., longer than the visual span). Therefore, cognitive-unit-based reading can be seen as a new, and arguably better candidate for explaining eye movement during reading.

The evaluation results are also consistent between English and Dutch (Figures 2–4; Tables 3, 4), which shows the validity of the models and the theory of cognitive-unit-based eye movement are not limited to a particular language. To collect more evidence of whether they are indeed language-independent, it would be interesting to run the same study on Chinese, where we may find even better results because there are no perceptual cues (spaces) that guide eye-movements in addition to the cognitive units. However, there is currently no publicly available large-scale Chinese eye-tracking corpus.

It must be noted that the perceptual span of our eyes is limited, so a cognitive unit should get more fixations when it exceeds the span. Perceptual span is not of concern to any segmentation model, but we can examine perceptual span anyway by limiting the unit length in the LiB model. If the maximum unit length is shorter than the real perceptual span, the predicted fixation location would be biased to the left for the cognitive units whose length are between the limitation and the perceptual span; if the maximum unit length is longer than the real perceptual span, the prediction would be biased to the right for the cognitive units whose length exceeds the perceptual span; the optimal maximum unit length should reflect the best fixation prediction. The results did show the best prediction scores when we limit the unit length to 16 in the English prediction task and to 13 in the Dutch prediction task (Figure 4), which is close to the finding that the perceptual span extends to 14–15 letter spaces to the right of fixation (Rayner, 1998).

This finding does not mean that there really is a maximum unit length in cognition. The current study does not aim to improve the prediction of eye fixations: the eye-fixation prediction task in this study only serves to cognitively evaluate the units segmented by the models. For this reason, we needed to prevent any prior information about eye movements (e.g., oculomotor constraints or linguistic knowledge) from “contaminating” the eye-fixation prediction task, which is why we did not include such prior knowledge in the models. However, in possible future work which explicitly aims to predict or explain the eye fixations, we may include the constraints from the physiological system and the linguistic attributes of reading material in the linking hypothesis between segmentation and eye-fixation. For example, the attention distribution in the perceptual span is actually asymmetrical (Reilly and Radach, 2006), which causes the optimal viewing position and preferred viewing location to be slightly to the left of the middle of a token (Rayner, 1979; McConkie et al., 1988) rather than the exact center as we assume in this study. If the fixation fails to land on the ideal location in a token, it will trigger an immediate re-fixation for correction (Nuthmann et al., 2005). Another example is that high-level linguistic attributes of tokens can also influence eye fixation by mediating the tokens' predictability (Ehrlich and Rayner, 1981; Balota et al., 1985; Warren and McConnell, 2007).

The concept of cognitive unit does have some connections to models of eye movement control in reading. The E-Z reader model assumes that the attention of reading shifts word by word, so it is categorized as a “serial attention shift” (SAS) model (Engbert et al., 2005; Reichle et al., 2009). An important alternative model is the SWIFT model, which assumes that parallel activation of multiple words over the fixated region is also possible. This model is categorized as a “gradient by attention guidance” (GAG) model (Engbert et al., 2005; Reichle et al., 2009). Although E-Z reader and SWIFT are still word-based models, so neither supports the processing of multi-word groups as single units, the latter model shares with the LiB model the idea that multiple words can be activated in parallel. Besides, human processing of cognitive units may involve two stages (familiarity detection and recognition; Yang et al., 2020a) which are similar to the two stages (familiarity check and lexical access) assumed by the E-Z reader model (Reichle et al., 2009).

### 4.2. Cognitive Units From Different Models/Motivations

We have seen the advantage of cognitive-unit-based predictions for explaining eye fixation during reading (Table 3). The question then turns to which model best segments the cognitive units from text, that is, which model more accurately predicts the eye fixations. The answer is also in Table 3: LiB is on par with AG-collocation, AG-word performs the worst, and CBL is in between. Moreover, LiB (unlike AG-word and AG-collocation) predicts longer fixation distances on Dutch than on English, in accordance with the observed pattern (Figure 2). The performance differences between the models may reflect differences between *how our cognition defines the units* and *how the model defines the units*.

The CBL model follows the notion that both children and adults can learn multi-word sequences from words, and that learning is based on the transitional probabilities (McCauley and Christiansen, 2017). The units in CBL are words and multi-word sequences, not subwords. However, McCauley and Christiansen (2019) also admit that learning directly from individual words is unrealistic for children. In addition, both CBL and LiB tend to memorize frequent units, but only LiB also forgets the units that are frequent but increase the numbers of types or tokens and therefore violate the principle of least effort. Thus, in the current study, the higher performance of LiB over CBL may be attributed to the character-based learning and the principle of least effort.

The learning in AG takes another approach: it tries to infer the *optimal* (in the Bayesian view) tree structures to represent the given language material (Johnson, 2008). The model clusters the symbols in the corpus in a hierarchical way so its output units are shown at the middle level(s) of the hierarchy (Johnson and Goldwater, 2009). The similar performance of AG-collocation and LiB (Table 3) arises in spite of their very different structures and workflows. Although AG is based on Bayesian estimation whereas LiB is based on cognitive assumptions, both models aim to optimize the lexicon and the segmentation, and thereby learn supra-word and subword units (Figure 3), which may result in their similar performance. Another interesting phenomenon is that the AG-collocation units show much better performance than the AG-word units in the fixation prediction task (Table 3). Since AG-word finds only very few supraword units and many subword units (Figure 3), the higher performance of AG-collocation and LiB suggests that language cognition prefers larger units.

The motivation underlying the LiB model is the least-effort principle: LiB regards the text chunks fitting the least-effort requirement as the cognitive units during reading. This motivation follows William of Ockham's (1287–1347) law of parsimony, which is also known as *Occam's razor*. The law of parsimony for cognition is applicable since cognitive resources are limited. This motivation also follows Zipf's (1949) argument that all human behavior can be systematized under the Principle of Least Effort (PLE). Although neither Occam's razor nor PLE is tangible and quantifiable enough for a computational model, LiB implements their philosophy by interpreting least effort (in language processing) as less use of both working memory (the number of cognitive unit tokens) and long-term memory (the number of cognitive unit types).

The balance between working and long-term memory can be seen as the balance between computation and storage, which is still under debate. Chomsky and Halle (1968) believed complex words are generated from simpler forms. Baayen (2007) criticized this generative theory, because in that case the balance of storage and computation is shifted totally to the maximization of computation and the minimization of storage. He, in turn, claimed the importance of storage, but did not provide a measure of the two. Minimum description length (MDL; Rissanen, 1978) fills the blank to some extent: MDL describes both storage and computation by their required encoding bits and so MDL unifies the two parts. Yang et al. (2020b) showed that LiB also minimizes description length of a corpus compared to some other models. MDL assigns storage and computation the same weights. However, they are in different cognitive systems (long-term memory vs. working memory) and may have different cognitive processing costs. These costs may also depend on individual differences. In the LiB model, these differences can be reflected in hyperparameters.

Moreover, the cognitive units should be generalizable if we want them to be practical. The reading experience of an educated adult relies to a large extent on language materials. It is meaningless if the language users learn the cognitive units from some piece of language materials but cannot use them on new material. Fortunately, a task-independent but large-scale corpus can help to discover cognitive units that are at least as usable as those from the task-specific corpus (Table 4). This finding demonstrates the training generalizability of the segmentation models and the external validity of the trained cognitive units. Besides the better performance, it is also worth noting that the time and memory costs of LiB on large training data are reasonable because LiB only requires simple computations (compared with Bayesian computation) and a small lexicon (compared with tracking all unit frequencies or even bigram transitional probabilities). The saving of time and storage suggest that the LiB lexicon is in itself actively trying to optimize toward a saturation point, or to converge toward a set of *good* cognitive units.

### 4.3. Room for Improvement of Cognitive Unit Discovery

The ability to predict eye fixations demonstrates the cognitive reality of the concept *cognitive unit*, but cognitive units can do more than predict the eye fixations. Those units by definition are the building blocks of human language processing. They may serve as better operational units in computational linguistics, psycholinguistics, language education, translation, and so on. As an example in computational linguistics, the corpus segmented into LiB's cognitive units shows more concise description and lower N-gram language model perplexity than when words form the units (Yang et al., 2020b). All in all, it is still worth seeking ways to improve the discovery of cognitive units.

Although the hyperparameters for training LiB in the current study had almost the same values as in a previous LiB study (Yang et al., 2020b), which is unrelated to eye-fixation prediction and thereby avoids the double-dipping issue, we still want to decouple LiB from its hyperparameters to discover the cognitive units shared by most users of a language or the cognitive units that reflect the shared thoughts in multiple languages. CBL is an exemplar of such decoupling because it has no hyperparameters and its built-in parameter (the frequency threshold for constructing a chunk) is adjusted according to the running average of the chunk frequencies. We intend to also make the hyperparameters adaptive in the future LiB model. Alternatively, we may aim to make LiB into a dissipative system (a system that can reach a steady state when it interacts with the environment), more self-organized and insensitive to the initial hyperparameters.

Decoupling LiB from its hyperparameters enhances the generality of the model. On the opposite side, the model can be tuned specifically to simulate the individual properties of a human agent; for example, the unique lexicon of a person with aphasia, or the change of a child's mental lexicon during language acquisition. Introducing more hyperparameters related to individual cognitive differences may help to discover idiosyncractic cognitive units. Possible relevant hyperparameters could be the perceptual span and the balance between long-term memory and working memory that we have discussed above, and other empirical knowledge of physiology.

Lastly, we should note that the prediction scores of different models vary within a narrow range. Also, altering training material from GECO to 100 times larger corpora did not lead to an F1-score improvement of more than two percentage points. The reason for the apparent performance ceiling could be that the current LiB model, as well as the CBL model and the AG model, discover only the frequent units. Some infrequent units can also be cognitive units: for example, people may immediately memorize the name of a never-heard city in a breaking news since the name is salient in the context. The current LiB model is not sensitive to such contextual semantic and pragmatic information.

## 5. Conclusion

The current study demonstrates the advantage of cognitive-unit-based reading theories over traditional word-based reading theories by using an eye-fixation prediction task. Among the computational implementations of cognitive-unit-based reading as unsupervised word segmentation, the LiB model shows good performance and high efficiency, and indicates that least effort in both working memory and long-term memory may play an important role during language learning and processing. Overall, the study supports the theory that the mental lexicon stores not only words but also smaller and larger units, suggests that fixation locations during reading depend on these units, and shows that unsupervised segmentation models can discover these units.

## Data Availability Statement

The original contributions presented in the study are included in the article. All code and datasets involved in modeling and experimentation are available at https://github.com/ray306/LiB-predicts-eye-fixations. Further inquiries can be directed to the corresponding author/s.

## Author Contributions

JY designed and programmed the model and performed the analysis. JY and SF designed the analysis. JY, SF, and AB wrote the manuscript. All authors contributed to the article and approved the submitted version.

## Funding

This work was funded by an International Max Planck Research School for Language Sciences Ph.D. fellowship awarded to JY (grant period 2017–2021).

## Conflict of Interest

The authors declare that the research was conducted in the absence of any commercial or financial relationships that could be construed as a potential conflict of interest.

## Publisher's Note

All claims expressed in this article are solely those of the authors and do not necessarily represent those of their affiliated organizations, or those of the publisher, the editors and the reviewers. Any product that may be evaluated in this article, or claim that may be made by its manufacturer, is not guaranteed or endorsed by the publisher.

## Footnotes

^1^https://expsy.ugent.be/downloads/geco/.

^2^https://www.corpusdata.org/coca/samples/coca-samples-db.zip.

^3^The metric is the product of log_2_|lexicon| (the cost of storing unit types) and $\frac{1}{\text{average chunk length}}$ (the cost of computing unit tokens).

^4^To simplify the model, we removed a regularizer that only memorizes the chunk tokens that appear more than twice in a single epoch in the LiB model described by Yang et al. (2020b). We reduced the *probation period* to an arbitrary value of 3, since the original setting will cause the lexicon to grow too fast after removing the regularizer.

^5^https://github.com/ray306/LiB-predicts-eye-fixations/tree/main/other_models/cbl_modified.py.

^6^https://github.com/ray306/LiB-predicts-eye-fixations/tree/main/other_models/AdapterGrammar/ag_geco/Makefile.

^7^https://scikit-learn.org/stable/modules/generated/sklearn.metrics.f1_score.html.

^8^The training of the AG model on large scale corpus is not feasible because of its very low time efficiency (more than 10 h on GECO).
